# The Physiology of Man

**Published:** 1867-11

**Authors:** 


The Physiology of Man. Designed to represent the existing state of Physiological Science, as applied to the Functions of the Human Body. By Austin Flint, Jr., M.D., Professor of Physiology and Microscopy in the Bellevue Hospital Medical College, and in the Long Island College Hospital; Fellow of the New York Academy of Medicine, etc., etc. Alimentation; Digestion; Absorption; Lymph; and Chyle. New York: D. Appleton & Co. 1867.

This volume constitutes the second of a series, and, like the first, is published in superior style. It contains 556 pages, and affords the reader a very full and instructive review of the state of knowledge concerning the great functions of Alimentation, Digestion, Absorption, Lymph, and Chyle. The first volume related to the Blood, Circulation, and Respiration. Each volume is complete in itself, and supplied with a copious index.

The author is performing a work of real merit, and should be encouraged by the liberal patronage of the profession. The present volume can be found at S. C. GRIGGS & Co., 41 Lake Street. Price $4.50

---

Medical Uses of Electricity, with special reference to General Electrization as a Tonic in Neuralgia, Rheumatism, Dyspepsia, Chorea, Paralysis, and other affections associated with general debility. With illustrative cases. By GEO. M. BEARD, M.D., and A. D. ROCKWELL, M.D. New York: WM. WOOD & Co., 61 Walker Street. 1867.

This is a very neatly printed and bound duodecimo volume of 65 pages. The principal part of the matter was originally contained in a series of articles published in the *New York Medical Record;* but both authors and publishers have conferred a favor on the profession by giving it a more permanent and generally accessible form. The chief object of the authors is, to illustrate the *superior efficacy of general electrization* over the more common practice of applying it locally. Practitioners will find this little monograph worthy of a careful perusal.

Price.—Full-bound, $1.00; in paper, $0.75.

---

Is It I? A Book for Every Man. A Companion to Why Not? By HORATIO ROBINSON STORER, M.D., of Boston, Vice-President of the American Medical Association. Boston: LEE & SHEPARD. 1867.

This is a duodecimo volume of 154 pages. The subjects treated of are:

1. It is not good to be alone.
2. Marriage as a Sanitary Measure.
3. How early in life is marriage to be advised?
4. The Rights of the Husband.
5. Are these Rights Absolute, or Reciprocal with Duties?
6. Should mere Instinct, or Reason, be the rule?
7. Arguments and Counter-Arguments as to Divorce.
8. A Plea for Woman.

These important topics are discussed by the author, in such

